# Conference Calendar

**DOI:** 10.1002/cfg.315

**Published:** 2003-07


					Comparative and Functional Genomics
Comp Funct Genom 2003; 4: 447-449.
Published online in Wiley InterScience (www.interscience.wiley.com). DOI: 10.1002/cfg.315
Conference Calendar
November 2003-January 2004
Animal models
CSHL -- Rat Genomics and Models, 11-14
December 2003
http://meetings.cshl.org/2003/2003meetings.htm
KEY -- Natural Variation and Quantitative
Genetics in Model Organisms, 8-13
January 2004
http://www.symposia.com/Meetings/View-
Meetings.cfm?MeetingID=671
Bacteria
ASM -- Biofilms 2003, 1-7 November 2003
http://www.asmusa.org/mtgsrc/biofilms2003.
htm
Bioinformatics
4th Georgia Tech and University of Georgia
International Conference on Bioinformatics. In
Silico Biology: Biological Networks -- from
Genomics to Epidemiology, 13-16
November 2003
http://opal.biology.gatech.edu/GeneMark/
conference/
ESF IAFG -- Advanced Data Mining and
Visualization Approaches to Systems Biology,
13-15 November 2003
http://ijsr32.infj.ulst.ac.uk/e10110731/
ESF2003.htm
GIW 2003 -- 14th International Conference
on Genome Informatics, 14-17
December 2003
http://giw.ims.u-tokyo.ac.jp/giw2003/
WSEAS -- Mathematics and Computers in
Biology and Chemistry, 19-21 December 2003
http://www.worldses.org/conferences/2003/tene-
rife/mcbc/index.html
3rd IEEE International Conference on Data
Mining, 19-22 November 2003
http://www.cs.uvm.edu/xwu/icdm-03.html
Pacific Symposium on Biocomputing (PSB
2004), 6-10 January 2004
http://psb.stanford.edu/
Evolution/comparative genomics
Wellcome Trust Comparative & Functional
Genomics Workshop, 2-5 November 2003
http://www.wellcome.ac.uk/hinxton/cfg
SICB -- SICB 2003 Annual Meeting, 4-8
January 2004
http://www.sicb.org/meetings/2003/index.php3
Functional genomics
IBC -- RNAi: Breaking through the Target
Validation and In Vivo Delivery Bottlenecks,
3-4 November 2003
http://www.lifesciencesinfo.com/RNAi
ESF IAFG -- RNAi: the Technology to
Revolutionize Functional Genomic Research:
What Are the Limitations? 20-22
November 2003
http://www.functionalgenomics.org.uk/sections/
activities/workshops.htm#Meyer
Copyright  2003 John Wiley & Sons, Ltd.
448 Conference Calendar
General
43rd Annual Meeting of the American Society
for Cell Biology, 13-17 December 2003
http://www.ascb.org/meetings/am2003/main03-
mtg.htm
KEY -- Emerging Mechanisms of Epigenetic
Regulation, 21-26 January 2004
http://www.symposia.com/Meetings/View-
Meetings.cfm?MeetingID=700
Multigenomes
Plant and Animal Genome XII Conference,
10-14 January 2004
http://www.intl-pag.org/
Pathways and metabolomics
CHI -- Metabolic Profiling, 8-10
December 2003
http://www.healthtech.com/2003/mbp/index.htm
Pharmacogenomics
Future Challenges for Target Validation, 3-4
November 2003
http://www.marcusevans.com/EVENTS/Life
Science CFEventinfo.asp?RecID=7705
Biomedical Research Applications in Drug
Discovery Technology (Asia-Pacific), 3-5
November 2003
http://www.drugdisc.com/asiapacific/
6th International Meeting on SNPs and
Complex Genome Analysis, 20-23
November 2003
http://snp2003.nci.nih.gov/snp2003/home.cfm
KEY -- Human Genome Sequence Variation
and the Inherited Basis of Common Disease,
8-13 January 2004
http://www.symposia.com/Meetings/View-
Meetings.cfm?MeetingID=670
KEY -- How Will Genomic Biomarkers Impact
Drug Discovery and Clinical Practice? 26-30
January 2004
http://www.symposia.com/Meetings/View-
Meetings.cfm?MeetingID=705
Plants
2nd International Nutrigenomics Conference,
6-7 November 2003
http://www.bastiaanse-communication.com/9/9.
html
BSPP -- Presidential Meeting 2003: Plant
Pathogen Genomics -- from Sequence to
Application, 15-18 December 2003
http://www.bspp.org.uk/meetings/bspppresprog-
2003.htm
Proteomics
Understanding Biological Systems through
Proteomics, 2-4 December 2003
http://sps03.fontismedia.com/
CHI -- PEPTALK: The Protein Information
Week, 12-15 January 2004
http://www.chi-peptalk.com/
Systems biology
BioSilico 2003: The Strategic Summit on In
Silico Biology, 23-24 October 2003
http://www.bioedge.net
4th International Conference on Systems
Biology (ICSB), 5-9 November 2003
http://icsb2003.molecool.wustl.edu/
Transcriptomics
4th International Conference for the Critical
Assessment of Microarray Data Analysis
(CAMDA 2003), 12-14 November 2003
http://camda.duke.edu/camda03/
Copyright  2003 John Wiley & Sons, Ltd. Comp Funct Genom 2003; 4: 447-449.
Conference Calendar 449
Biotech industry
SRI -- Pharma and Biotech Licensing and
Deal-making Summit, 22-23 January 2004
http://www.srinstitute.com/part iter site page.
cfm?iteration id =567
Key
ASM: American Society for Microbiology: http:
//www.asmusa.org
BSPP: British Society for Plant Pathology: htpp:
//www.bspp.org.uk/
CHI: Cambridge Healthtech Institute: http://www.
healthtech.com
CSHL: Cold Spring Harbor Laboratories: http:
//meetings.cshl.org/index.htm
ESF IAFG: European Science Foundation -- Inte-
grated Approaches for Functional Genomics: http:
//www.functionalgenomics.org.uk/
IBC: IBC Conferences: http://www.ibc-lifesci.
com
IEEE: Institute of Electrical and Electronics Engi-
neers: http://www.ieee.org/
KEY: Keystone Symposia: http://www.symposia.
com/
SICB: The Society for Integrative and Comparative
Biology: http://www.sicb.org/
SRI: Strategic Research Institute: http://www.srin-
stitute.com/
WSEAS: World Scientific and Engineering Aca-
demy and Society: http://www.wseas.org
Copyright  2003 John Wiley & Sons, Ltd. Comp Funct Genom 2003; 4: 447-449.

				

